# What is the extension of the extended mind?

**DOI:** 10.1007/s11229-015-0799-9

**Published:** 2015-06-29

**Authors:** Hajo Greif

**Affiliations:** 1 MCTS, Technische Universität München, Munich, Germany; 2 Department of Philosophy, Alpen-Adria-Universität, Klagenfurt, Austria

**Keywords:** Extended cognition, Proper functions, Niche construction, Developmental systems, Cognitive integration, Ecological psychology

## Abstract

Two aspects of cognitive coupling, as brought forward in the Extended Mind Hypothesis, are discussed in this paper: (1) how shall the functional coupling between the organism and some entity in his environment be spelled out in detail? (2) What are the paradigmatic external entities to enter into that coupling? These two related questions are best answered in the light of an aetiological variety of functionalist argument that adds historical depth to the “active externalism” promoted by Clark and Chalmers and helps to counter some of the core criticisms levelled against this view. Under additional reference to conceptual parallels between the Extended Mind Hypothesis and a set of heterodox theories in biology—environmental constructivism, niche construction, developmental systems theory—an argument for the grounding of environmentally extended cognitive traits in evolved biological functions is developed. In a spirit that seeks to integrate extended functionalism with views from cognitive integration and complementarity, it is argued (ad 1) that instances of environmental coupling should be understood as being constitutive to cognitive functions in either of two distinct ways. It is further argued (ad 2) that the historically and systematically prior environmental counterparts in that coupling are features of the natural environment. Language and linguistically imbued artefacts are likely to have descended from more basic relations that have an extension over the environment.

## Introduction

In their brief, bold, and controversial manifesto of a thorough, “active” externalism in the philosophy of mind, titled “The Extended Mind”, Clark and Chalmers highlight “the active role of the environment in driving cognitive processes” (1998, p. 7). Not only does the content of mental processes or linguistic expressions depend on what is in the environment—as it does according to externalist views in the philosophy of mind and language, respectively.^1^ In some respects at least, the very accomplishment of cognitive processes, and, in a relevant subset of cases, their very existence, depends on what is in the environment. Clark and Chalmers argue that there are situations in which “the human organism is linked with an external entity in a two-way interaction, creating a *coupled system* that can be seen as a cognitive system in its own right” (1998, p. 8, emphasis in original).

This externalist position, in its general outlook, shall be endorsed here. However, the notion of cognitive extension has become many things to many people in a continuously sprawling debate, where the most forceful critiques appear to frequently miss the most serious points that arguments for extended cognition are capable of making.^2^

On pain of adding another specimen to the zoo of views what cognitive extensions really are, I shall carve out an interpretation that strengthens the functionalist credentials of the Extended Mind Hypothesis by rooting it in natural history. My guiding questions will be these: firstly, how shall the functional coupling between the organism and some entity in his environment be spelled out in detail? Secondly, what are the paradigmatic external entities to enter into that coupling? In seeking to answer these questions, I will make a suggestion towards sharpening the notion of “extension” that is (or should be) in play here: the paradigm of cognitive extensions are those features of the environment which play either of two kinds of constitutive role to the function of some cognitive trait, and, in functional-historical terms, these features have been primarily natural rather than artificial. This suggestion shall help preventing the notion of cognitive extension from becoming extensionally bloated to the point of vacuousness.^3^

I will begin by placing some of the central claims of the Extended Mind Hypothesis—its notion of “active” versus “passive” externalism and its notion of features of the environment serving cognitive functions—in the context of aetiological theories of function and some positions within evolutionary biology (Sects. 2 and 3, respectively). The insights from this discussion will then be used to identify a number of possible interpretations of what extensions of the mind are, and what explanatory roles they might play in cognitive inquiries, with particular attention to the specific ways in which features of the environment may be constitutive to cognitive functions (Sect. 4). The constitutiveness theme will be further discussed in Sect. 5, with the aim of providing some common ground for “parity” and “complementarity” views of extendedness. Finally, I will discuss two paradigms of cognitive extension in the light of the aetiological variety of functionalist argument developed in the preceding sections (Sect. 6).

## The extension of functional histories

When locating themselves within the context of contemporary philosophical debates, Clark and Chalmers (1998, p. 9) characterise active externalism as “active” for its focus on interactions between organism and environment in the here and now. The activity of interest is in the environment and the organism at the same instance, and it is that concurrent activity which serves to make both cognition extended and externalism active. Conversely, Clark and Chalmers characterise traditional semantic externalism as “passive” for its focus on the causal history that supposedly endows linguistic items, and by extension of the argument (McGinn 1977), mental events with their meanings. It is this history of interactions that explains any possible difference between the contents of two prima facie identical mental or linguistic tokens, no matter what the current interactions may look like to participants and observers.

Hence, if one takes active externalism by its ahistorical first word, the chemical difference between water and twin water in Putnam’s well-known thought experiment (1975a), contrary to what its author aimed to demonstrate, will *not* matter as long as my interaction with either substance produces identical effects, cognitively and practically. Conversely, on the “passive” account, the different histories of earthly and twin-earthly water are all that matters in making the difference between the semantic success conditions for “water” tokens being applied to either substance, even if I cannot see, feel or taste any difference between the two.

Although the active/passive terminology is now well-established (see, for example, Carter et al. 2014), there remains a certain artificiality to referring to the varieties of externalism in this way. Past interactions have been relevant to shaping the content of some present linguistic or mental token, and there is no reason to assume the past to be in any normative sense remote from the present. What Clark and Chalmers are after is quite different from Putnam-style semantic externalism though: their focus is on the locus of cognitive processes, whereas Putnam, Burge, McGinn and many others are concerned with the external conditions that ground the content of mental or linguistic tokens. The difference at issue here is also known as “vehicle” versus “content” externalism (see Hurley 1998, 2010). On an ahistorical reading of active externalism, the concrete content of what is being processed appears secondary to the issue of the locus of cognitive activity, just as history is deemed secondary to present states of cognitive affairs.

Such a choice of priorities might be unfortunate, for several interrelated reasons—of which, to be sure, theories of extended cognition have become at least partly aware since that initial statement.^4^ Nonetheless, it will be worthwhile to spell out why, in terms of a unified rationale, any serious theory of extended cognition will benefit from accounting for the history of the functions of environmental coupling.

Firstly, when referring to the functions of some object or process, one will have to tell the difference between genuine functions and coincidental effects. That difference can only be established on the grounds of a history of how the object or process in question came to display the effects that it displays—and why it fails to do so under some circumstances. For artefacts, their design will provide part of the relevant criteria, whereas all relevant criteria will be historical in the case of natural systems and their functions.

Secondly, the content of cognitive activities—what in the environment they refer to and whether, why and how they succeed in doing so—is relevant to an explanation of the shape, function and extension of these activities, historically and at present, on more than one level. On the one hand, the concrete content of some cognitive process depends on the history of cognisers living in a concrete natural and social environment to which they relate. More fundamentally, on the other hand, the mechanisms for producing and acting upon these contents are rooted in histories of evolution and ontogenetic development, broadly construed.

Thirdly, present interactions dynamically reshape the mode of extension in many cases. There is no ending to history. If the conditions under which some relation of cognitive coupling is realised are altered—either from within or from outside the organism—that change, unless ending in failure of the entire process, will provide some degree of variation that will result in a modified coupling relation further down the line. Variability in environmental conditions will be one key factor in shaping coupling relations.

The common rationale to unify this set of claims is an aetiological, that is, historically based variety of functionalist argument. It departs from the classical Putnamian point of “machine state functionalism”, which is based on the assumption that one and the same function may be realised in a variety of different structures (Putnam 1975b). This claim of multiple realisability has been central to any argument for the possibility of Artificial Intelligence, and it is equally important to the “parity principle” in the Extended Mind debates. It has its roots in Turing’s observation that any logical or mathematical operation that is computable at all can be realised by a set of elementary formal operations, which may in turn be implemented in a broad, in principle indefinite, variety of physical systems (1936). The claim of multiple realisability is also implied by observations on neuronal equipotentiality, according to which one cognitive or perceptual function can be realised by a variety of neuroanatomical structures (see, for example, the discussion of Karl Lashley’s work in Proust 1995).

While sharing with these types of functionalism the basic notion of a relation of underdetermination between functions and structural properties of some system, an aetiological account is interested in the concrete enabling and constraining conditions under which functions come to be established and realised. Functional aetiologies have their natural home in natural history and the Darwinian theory of evolution. Charles Darwin gave an elegant interpretation to the observation made by many naturalists that commonalities in form (“homologies”) and commonalities in function (“analogies”) might be, partly or wholly, disjunct between species of different taxa.^5^ On the one hand, one ancestral form, for example a mammal’s forelegs, may come to serve divergent functions—as wings in bats and arms in human beings. On the other hand, one and the same function may come to be accomplished by structures of various degrees of similarity that have developed along independent pathways in genealogically remote species—as for the structurally similar but evolutionary independent lensed eyes in vertebrates and many Cephalopods, and the structurally dissimilar and equally independent compound eyes in Arthropoda. Accordingly, the concrete histories of convergent and divergent functions within and between populations will matter to an aetiological account.

If cognitive traits have biological functions in the same way as other traits of an organism have biological functions, one will be entitled to analyse the mechanisms that realise these processes in the same functional-historical terms. Like any other trait, they will be subject to processes of variation and natural selection. In turn, the content of these processes can be analysed in analogous fashion, to the extent that a type of cognitive state of one or a number of related individuals is constituted by reproducible tokens that may succeed or fail to map onto some world affair, and hence be selected to accomplish that mapping. These are the basic assumptions of what has become known as “teleosemantics” or “teleofunctionalism”, as first and most influentially introduced by Millikan (1984).^6^

More precisely, a functional-historical account of some cognitive trait will recur to the contributions that the effects of that trait have made with sufficient frequency among its ancestral bearers to their rate of reproduction as compared to other members of the same population who did not possess that trait. By virtue of conferring a reproductive advantage over the course of several generations, the trait will acquire the *proper function* of producing these effects.^7^ The “ancestors” and “generations”, rather than being organisms themselves, may also be component mechanisms, and they may also be reproducible forms that can be iteratively used by some individual or a collective thereof, such as artefacts or linguistic items. The individual tokens of some such reproducible item have proper functions derived from the functions of the mechanisms that produce them. It is the direct proper function of those mechanisms to generate the kind of effects in question if these effects, qua individually bearing certain relations to the environment with sufficient reliability, form part of the necessary conditions for the reproduction of the mechanisms themselves and of the systems that rely on their presence. In turn, the tokens of some reproducible item have the derived proper function of being adapted to some concrete world affair if and when one relevant condition for their reproduction lies in their co-occurrence with that sort of world affair and, in a relevant subset of cases, their role in directing an organism’s behaviours towards it.

Nothing in this account rules out the possibility of mechanisms that are only partly based within an organism. In fact, Millikan (1993, p. 179) herself considers the insight that “the organismic process has no skin” fundamental to the science of psychology.^8^ Since all sorts of traits, cognitive and other, are characterised in terms of their historically acquired functions, and since that history incorporates whatever turns out to realise the function in question, the locus of the components involved in realising it is systematically irrelevant, under a two-part proviso: the organismic traits involved are to be coupled with persistent or reproducible features of their environment with a degree of reliability sufficient for meeting the reproduction criterion, where that coupling occurs in accordance with a uniform explanation that accounts for the “normal mechanisms” of coupling. Hence, although the content externalism defended in the aetiological account does not entail a vehicle externalism, it is methodically impartial to the locus of the vehicles involved.

As to the concrete benefits of an aetiological perspective for extended functionalism, accounting for the historical nature of functions will, firstly, provide an argument against the “coupling/constitution” objection brought forward by Adams and Aizawa (2001): in contrast to what the critics suspect, instances of accidental coupling between an organism and some entities in his environment will not count as coupling proper. Nor will legitimate instances of coupling endow the individual entities thus coupled with the characteristics of the entire coupled system. These entities play constitutive, historically established roles in the establishment or accomplishment of the functions of the entire system, where these roles may widely diverge. I will discuss this point in more detail in Sect. 4.

Secondly, and for related reasons, accounting for the historical nature of functions will provide an argument against the “fleeting vs. persistence” criticism that has been levelled against extended cognition, first and foremost, by Rupert (2010). The suspicion here is that environmental extensions, unlike internally based cognitive capacities, are not persistently coupled with the organism. If some such extension is accidentally detached from the cognising organism, it seems, the cognitive system comes apart, only to be re-instantiated if and when that extension is successfully recovered. Clark’s reply to this charge was to emphasise the conditions of persistence and reliability (“glue and trust”), which supposedly rule out overly ephemeral coupling relations (Clark 2010a, p. 83 f). If the coupling in question has a historically established function, the issue of temporary non-occurrence or non-performance of that coupling relation, rather than being resolved by means of such auxiliary conditions, is actually *dis*solved: it does not matter if the function in question, for temporary loss of one component, turns out not to be performed in many instances, as long as the coupling occurs with a degree of reliability and according to an unequivocal regularity so as to become part of the explanation for the reproduction of the organic and, in some cases, the environmental components involved. To use a non-cognitive case of extension for comparison (see Menary 2010b, p. 14): it does not matter if a spider loses her web once in a while, or perhaps even very often, as long as the, however intermittent, production and use of webs provides part of the explanation for the presence of web-making mechanisms in the spider population.

Thirdly and most profoundly perhaps, a historical account of functions will counter Sprevak’s critique of classical machine-state functionalism (2009) which targets the “Martian intuition” that an identical or analogous kind of function can be realised by physically or biologically very differently constituted systems. On that intuition, which is basically the Lewisian one (1980), if we can specify a set of functions for certain traits and behaviours in human beings *and* Martians, we should be able in principle to identify which concrete structures within or around the human being or Martian will play what sort of causal role in accomplishing those functions. However, Sprevak objects, one could easily imagine the conditions of persistence and reliability of the respective structures and the coupling relations involved to be violated in manifold ways, and hence fail to meet the specifications laid out by extended functionalism. Playing on the Martian intuition in this way will probably kill the Martian—but, if we follow the historical account of functions, it will not kill the intuition. Any coupling with whatever structure that were as unreliable or otherwise insufficient or even freakish in nature as described by Sprevak would militate against the imagined organism’s reproductive chances, and against the factual (rather than conceptual) possibility of his existence. Conversely, Sprevak’s Martian may be imagined to be in command of capabilities and enter into environmental coupling relations so unfamiliar that we could not find any remote analogue in the specifications of human cognition. But then, the entire functional analogy will not hold. After all, there might be hyper-cognitive functions to Martians that human beings are insufficiently equipped to imagine in the first place. At any rate, the historical account of functions serves as a reminder that observations of real properties of real members of real populations provide a more solid foundation to an inquiry into cognitive functions than counterfactual-supporting conditions in some nearby or remote possible worlds.

## Evolution, construction, and development

The reservations against history diagnosed in the previous section become explicit when Clark and Chalmers introduce a set of criteria that are supposed to be necessary and sufficient for the function of internal or external resources as components of cognitive processes (1998, p. 17; see also Clark 2010b):

1. The resource should be of reliable availability, and should be used on a frequent basis.
2. The information provided by that resource should be directly available.
3. This information should be endorsed automatically, i.e., without requiring further reflection on its reliability.
4. Present endorsement of this information should be based on conscious endorsement in the past.

The authors add a footnote to this list in which the implication of the constancy (3) and the past-endorsement (4) criteria that history is co-constitutive of cognitive processes is countered by the suggestion that the past-endorsement criterion might be dropped and the constancy criterion be given “a purely dispositional reading”: one is found simply to endorse the information automatically; it is not asked why. While this might be a sensible tactical manoeuvre in terms of catering for the broadest possible range of extensions of the mind, relativising the latter two criteria amounts to skirting an inquiry into the normal mechanisms of cognitive coupling. The past-endorsement criterion in particular would assimilate the argument to a teleofunctional one: the decisive condition for past endorsement being causally relevant to present endorsement is that the former has been successful, although it might also have been conscious. If endorsement had not been successful, although it might still have been conscious, there would be no endorsement at present.

Beyond tactical considerations, one possible systematic reason why the proponents of the Extended Mind Hypothesis have been reluctant to adopt a historical account of cognitive functions is detectable in Wheeler and Clark (2008)—a contribution that actually acknowledges arguments for the rooting of cognitive extensions in evolutionary history: the authors match the dyad of extended/embodied cognition and cultural evolution hypotheses against the tenets of evolutionary psychology. The latter is charged with a view of evolution that takes psychological and physiological adaptations alike to be, temporally remotely anchored, solutions to a predetermined set of problems posed by the environment, where these solutions will be uniformly distributed over a species while being expressly domain-specific. Moreover, Wheeler and Clark continue, the possibility of organisms modifying their environments to fit their specific needs is not considered by evolutionary psychologists. The basic concern here appears to be that an anchoring of cognitive extensions in past states of affairs that are beyond present cognisers’ reach—especially if the past endorsement criterion concerns the *evolutionary* past—would undermine the notion of a two-way interaction that is central to the coupled system claim (see p. 1 above). Adaptations and the conditions to which they respond might be more malleable and more specific to sub-populations in the here-and-now than evolutionary psychology appears to consider.

In order to get a clearer view of what is at issue here, a brief look at Dawkins’ theory of the extended phenotype (1999) will help. Notably, it is listed among the biological cousins of extended cognition by Wilson and Clark (2009). Prima facie, the parallels seem striking: Dawkins not only highlights the importance of an organism’s interaction with the environment but also promotes the notion of biological traits extending into, or incorporating features of, the environment. Dawkins suggests a change of perspective of inquiry under which the organism ultimately becomes “transparent” (1999, p. 4f, 250). What remains visible once the organism has become transparent are replicating gene sequences within a population and their interactions with their intra- or extra-somatic environment. By means of this, explicitly metaphorical, change of perspective, Dawkins argues that the organism might not always be the key unit to consider when explaining patterns of genetic replication. Instead, he suggests, an organism’s interaction with objects or other organisms within his environment will have to be counted into the explanation of whether and how a gene succeeds in replicating. Hence, we might, and often should, treat the organism and his environment in conjunction as the wider environment of some gene sequence in which it acts, and which it manipulates.

Precisely in rendering the organism thus transparent, Dawkins’ “gene’s eye view” re-instantiates the notion of a unitary, individual and individualistic agent who is clearly distinguished from his external environment, albeit with a substitution of the referent of “individual”. In many relevant cases, the manifest aims of individual organisms will end up subordinated to their genes’ hypothetical interests. Moreover, the systematic point of adopting this view within the context of evolutionary theorising is to highlight a possible, and perhaps the most relevant, unit of natural selection—as the main force in evolution and the source of any goal-directedness or progress therein. The effects that the organism’s own traits and behaviours might have on the course of evolutionary events will not figure prominently in such an account.

Wilson and Clark (2009) expressly endorse a continuum of theories in biology that takes issue with these implications of Dawkinsian view, and that bears more profound analogies to some of the central claims of extended cognition. That continuum encompasses, firstly, environmental constructivism as introduced by Lewontin (1982), secondly, the development of the latter into theories of niche construction, beginning with West and King (1987) and Odling-Smee (1988) and, thirdly, developmental systems theory, as developed by Oyama (2000). This continuum of theories is unified by its opposition to what has been termed by its critics (first by Gould and Lewontin 1979) the “adaptationist programme”, which in turn has Dawkins as one of its main advocates. For its emphasis on the nature of functions as, paradigmatically naturally, selected effects, and for its reliance on the positive selection *for* some trait, the teleofunctional account should be partly subsumable under the adaptationist rubric, too (these commitments are quite clearly stated in Millikan 2004, Chaps. 1–2).

Environmental construction, on the account presented in Lewontin (1982, 2000), will include any intervention by an organism or population into processes in the external environment that changes conditions therein in such a way that the intervention in question becomes a necessary part of the explanation of the nature of the organism or population and their adaptive success—or failure. The construction of environments is not to be understood in a straightforwardly literal sense, although the building of material structures might be involved. Environmental construction also includes cases where features of the environment remain physically unaltered while being treated in such a way as to affect the attainment of an organism’s or population’s goals. These two kinds of cases are distinguished by Godfrey-Smith (1996a, Chap. 5), in his critical reconstruction of Lewontin’s approach, as the *construction* of features of the environment in a more narrow sense, in terms of creating material structures and artefacts, versus the *constitution* of features of the environment, in terms of treating them in a certain way or putting oneself in a certain relation to them without materially changing the structure of the environment. (The use of the term “constitutive” by Godfrey-Smith will have to be distinguished from the notion of “constitutiveness” to be employed later in this essay.) Beaver dams and many other kinds of artefacts will be subsumed under the category of construction, activities of seeking out favourable conditions in space and time under the rubric of constitution. In either case, yet in different ways, organisms or populations are actively involved in determining the environmental conditions that are relevant to them.

The “construction” and the “constitution” types of cases, which are best viewed as complementary rather than mutually exclusive, should be distinguished from another type of intervention by organisms into environmental conditions that was also subsumed under the “construction” label by Lewontin: situations where the effects of an organism’s behaviours undermine rather than foster the attainment of his goals or his chances of reproduction. Such accidental side-effects only indirectly, and clearly only negatively, relate to the possible adaptive functions of the traits that produce those effects. If not in treating construction and constitution as equivalent, it is here where Lewontin’s notion of environmental construction becomes too broad to be entirely useful, as Godfrey-Smith observes.

Theories of niche construction, having found their paradigmatic formulations in Odling-Smee et al. (1996, 2003), may provide more focus to the notion of construction involved here. Broadly speaking, ecological niches are the specific sets of conditions in an environment that are reproductively relevant to a certain population. Different populations inhabiting one area at one time will depend on different sets of conditions at that place at that time. The make-up of these conditions partly depends on the activities of the organisms themselves. While some of the authors who introduced the concept of ecological niches into biology (especially Elton 1927) found the idea of a space of pre-existing ecological niches perfectly permissible, where some niche may be inhabited by different populations over space and time or even left uninhabited, there is no such thing as independently defined ecological niche for the niche constructivist. Niches are both made by and might be undone by the organisms inhabiting them. If there *is* some adaptive function to the construction and constitution of features of the environment, these features will become part of the necessary conditions in an explanation of an organism’s success on a proximate behavioural or an ultimate selective level, or both. If these features are indeed part of the necessary conditions in question, there are good reasons to view them as coupled with the organism: he has to track the presence of some feature and put himself in the right relation to it, or he has to modify some feature in order to prevail.

An approach that, among other strands of heterodox biological theorising, incorporates environmental and niche constructionism, is to be found in developmental systems theory (Oyama 2000). Being a deliberate change of perspective on the animate realm rather than a conventional predictive theory, as Oyama et al. (2001, 1f) admit in the introduction to their developmental systems anthology, it reverses the direction of change taken by Dawkins: a developmental system is the conjunction of organismic and environmental factors that accounts for the presence of certain phenotypic traits within an organism or population, where environmental and non-genetic organismic factors are considered as intrinsic to the development of the organism as genetic ones. On the one hand, an identical set of genes might be found in clearly distinct phenotypes. For example, first-generation worker ants of a newly founded colony, one of whose tasks is to construct many of the standard features of ant colonies, will look and behave differently from genetically identical later generation specimen raised in the fully established colony, and hence in an environment that was shaped by those first generations (Gordon 2001). Similarly, the sex of turtles and crocodiles is not genetically determined but depends on environmental temperature during embryonic development (Bateson 2001). On the other hand, modification of environmental factors may affect the development of traits that match with environmentally unmodified genetic variants, hence providing for distinct developmental routes to a similar phenotype, as in the phenomenon of “phenocopying” (Goldschmidt 1949).

Either way, the focus of inquiry has to be on the combined system, with environmental factors fully integrated but not necessarily interchangeable with genetic ones, and with the key unit of analysis often not being the individual organism but supra-individual entities. The very notion of one central unit of control in development might be misguided to begin with. Even mechanisms of inheritance might be distributed over a variety of factors, including persistent structures in the environment. There is no such thing as genetic information that could be taken by itself and still be informative about what phenotype an organism will develop. Such would be the case only if “strong instructionism” were true, that is, if genetic information were supposed to fully specify phenotypic traits (Wheeler and Clark 1999, 2008). Rather than being relegated to the status of context, however important, to the content of the genetic code, environmental and other non-genetic factors are placed on equal explanatory footing with respect to informing biological development. Some developmental systems theorists even reject the notion of genetic information altogether, and speak of genetic and non-genetic inheritance of developmental resources instead (Griffiths and Gray 2001).

The critique of adaptationism common to the biological allies of extended cognition can be unpacked into two more positively stated leitmotifs: a principled openness of both evolutionary and developmental pathways and an emphasis on supra-individual factors as explanantia, rather than explananda, for the respective theories. The principled openness is expressed, on a first level, in the marked abstention from postulating some unifying force that would govern phylogeny and ontogeny. Natural selection counts as just one among other relevant factors in evolution, and it works on various levels, from genes to populations. Genes are just one among other relevant factors in shaping organisms and, possibly, transmitting information between generations of a population. On a second level, there is no reason to assume that there is such a thing as evolutionary progress or adaptive optima. What might seem like a perfect adaptation now may turn out to be a disadvantageous solution later, and there is no way of knowing in advance. Current evolutionary states of affairs are historically contingent facts, providing no guidance as to how things should stand or where they will go next.

## The constitution of cognitive extensions

What do the observations in the two preceding sections tell us about the Extended Mind Hypothesis? After all, all parallels to the debates within evolutionary theory were only identified in retrospect. Nor did the Extended Mind Hypothesis, as originally conceived, relate to teleofunctional theories. Still, arguments from either tradition might help to elucidate, and possibly achieve, Clark and Chalmers’ explanatory aims, on two levels: the epistemological status of the hypothesis (discussed under the short form *Int*) and the question of the nature of extensions (discussed under the short form *Ext*).

### The status of epistemic claims

While the theories considered above partly figure as predictive theories in their respective scientific fields, developmental systems theory and the theory of extended phenotypes deliberately assume a different (separate or additional) role, namely as challenges to seemingly commonsensical perspectives on their subject matter. As such, they might ultimately but will not necessarily result in predictive theories.^9^ This dual notion of theories may serve as a template for a strong and a weak interpretation of the Extended Mind Hypothesis respectively: if, at least in important subclasses of cases, entities and processes in the environment are indispensable for the accomplishment or presence of some cognitive function, the expectation will be justified that the advocate of extended cognition shall identify the necessary conditions for those objects and processes to become part of that cognitive function while remaining impartial to what common sense might tell us. Some of Clark’s own works (in particular 2007) as well as some complementarity-based accounts (most notably Kiverstein and Farina 2011; Sutton 2010) aim in this direction, in looking for real-world cases where the assumption of environmental extendedness of cognitive processes accomplishes tangible explanatory tasks. If however it is a mere matter of perspective to view some objects in the environment as components of cognitive processes, the claim is considerably weaker: it amounts to a suggestion to temporarily suspend common sense beliefs in order to help us to a better understanding of some aspects of cognition.

The critique in Adams and Aizawa (2001), although not intending to capture this point, is diagnostic of what is at issue here. The authors explicitly mount a defence of common sense, presuming that common-sense views, *qua* being commonsensical, provide good guidance to cognitive inquiries. If, as Adams and Aizawa argue in alignment with a long-standing intellectual tradition (which includes, among many others, Popper 1959; Quine 1957), science is a methodical refinement and extension of common-sense reasoning, mobilising it against overambitious flights of imagination will be useful in itself. As, however, many scientific findings happen to defy common-sense beliefs, and as, arguably, the advancement of science sometimes even requires transgressions of the boundaries of common sense (as has been prominently argued by Einstein 1918, along with other physicists whose theories indeed defy common sense, as well as by philosophers as different as Nagel 1961; Feyerabend 1975), the force of this argument will be limited to begin with. If there are defensible reasons for adopting a counterintuitive image of how the world stands, and if evidence collected under the guidance of that image turns out to advance inquiry, the transgression will be vindicated. It will be difficult though to determine in advance whether that vindication is forthcoming. In terms of an epistemological economy, the Extended Mind Hypothesis incurs the risk of investing into a transgression of common sense whose returns are much less than certain.

### The nature of extensions

One common denominator of all theories discussed above is that there will be no way of determining the goals and accomplishments of an organism without in some way considering his coupling with the environment, where this coupling will have the nature of a two-way interaction rather than the organism either manipulating or being determined by the environment. Certain entities and conditions in the environment are relevant to the organism in such a way that he could not exist without them and that they would not exist without him, as the very set of relevant conditions that they are. On this set of views, the coupling between organism and environment plays a *constitutive* role to the trait or behaviour in question in either of two ways: without that coupling, it would not be possible at all, or it would have to rely on different means.

Organisms with a certain degree of adaptive plasticity, that is, the ability to respond in various and partly innovative ways to a given set of problems, can be found to alternate between using internal means and one or a variety of external objects or tools for the same purpose, each involving different organic and behavioural resources and, possibly, differential effects on reproductive chances. For example, geographically separate populations of wild chimpanzees (*Pan troglodytes*) have been observed to vary in using versus not using tools and in the kinds of tools they use for a certain purpose, in spite of identical biological constitution and ecological similarities between the respective populations’ habitats (Boesch and Boesch 1990; Boesch 1993; Matsuzawa 2001, Chap. 1). It has been suggested that cultural transmission of tool-using abilities is involved here.^10^ While there is an analogy of function in these cases, both the entities involved and the mechanisms by which they are integrated in pursuit of that function will be at variance. The constituents of the external and internal variants of the processes cannot be expected to be mutually substitutable. This is the *minimal* sense in which extensions may become constitutive to the accomplishment of some biological function. They will be called “constitutive$_{W}$” where required for disambiguation.

The situation will be different in cases where there are objects and artefacts in the environment that enter into some coupling without which a particular, and possibly vital, accomplishment for the organism could not be attained *at all*. Citing a famous example, without felling trees and constructing a dam from timber, stones and mud, so as to impede the flow of a creek and create a water reservoir, a beaver would neither be in the position to secure himself a sufficiently large territory for foraging nor to find a place for building lodges sufficiently protected from terrestrial predators (Dawkins 1999, pp. 200, 209). There is no ‘internal’ alternative for the beaver to building a dam. He does so because this is the only way available to him to secure the presence of some of the conditions on which he vitally depends. This is the *maximal* sense in which extensions may become constitutive to some biological function: extendedness will become essential to an explanation of the presence, rather than the mode of accomplishment, of some core functions—and, by implication, of the nature of the organisms to which those functions pertain. Cases of this kind will be labelled “constitutive$_{S}$”.

The constitutive$_{S}$ and constitutive$_{W}$ types of case are clearly distinct from an interpretation of cognitive extensions in which they appear as *instruments* of cognition: if the cognitive function in question could be equally performed by purely internal means, their role would be similar to that of an artefact that produces a behavioural output identical to that of some type of human action, and that does so on the basis of at least similar mechanisms. Internal and external elements would be mutually substitutable without any notable effects on the performance, and the mode of performance, of the cognitive function. If (and only if) extensions of the mind were supposed to be understood in such a straightforwardly instrumentalist fashion—which, to be sure, they are not—a central point of Adams and Aizawa’s critique of extended cognition (2001) would become tenable. The authors maintain that the causal processes involved in the use of cognitive extensions would have to be identical to those involved in internal or “intra-cranial” cognitive processes in order to count as functionally equivalent. Since the causal processes, as a matter of empirical fact, will be at variance, functional equivalence is not accomplished. If, however, the extensions Clark and Chalmers have in mind are not instrumental in nature but constitutive$_{W}$ to cognitive processes, the mechanisms involved in extended versus internally based cognitive processes can be freely acknowledged to be at variance. Adams and Aizawa hence appear to miss the entire point of functional analogy, which is precisely *not* determined on the grounds of a homology between the structures that realise some function but on the grounds of what purpose the respective structures serve.

### Varieties of extendedness

To sum up, we are presented with four different possible interpretations of the Extended Mind Hypothesis, on two partly interdependent levels:

$${ Ext}_{C}$$
The constitutiveness claim: entities in the environment are constitutive$_{W}$ or constitutive$_{S}$ to the function of some cognitive trait.
$${ Ext}_{I}$$
The instrumentalist claim: entities in the environment are instruments that are mutually substitutable with internally based cognitive traits.
$${ Int}_{S}$$
The strong interpretation: the Extended Mind Hypothesis shall identify part of the necessary conditions for the presence and function of the cognitive traits in question.
$${ Int}_{W}$$
The weak interpretation: the Extended Mind Hypothesis suggests some conditions under which cognitive processes are best viewed as including entities in the environment.

When introducing their hypothesis, what Clark and Chalmers appear to do is this: they present a set of theoretical claims that speak for an interpretation under which objects and conditions in the environment are part of the necessary conditions for the presence and function of some cognitive traits. Accordingly, the strong interpretation $({\textit{Int}}_{S})$ would seem in place here. However, these claims are quite casually placed alongside remarks on how a view of cognition as extended into the environment allows for a more elegant, unified and simplified explanation of cognitive processes (see Clark and Chalmers 1998, pp. 10, 14). There is no reference in these places to explanatory problems that would *require* a view of cognition as being thus extended. Accordingly, the weak interpretation $({\textit{Int}}_{W})$ would seem in place here.

There are two adjacent claims on the first pages of Clark and Chalmers’ essay where that ambiguity becomes manifest:

If, as we confront some task, a part of the world functions as a process which, *were it done in the head*, we would have no hesitation in recognizing as part of the cognitive process, then that part of the world is (so we claim) part of the cognitive process. (Clark and Chalmers 1998, p. 8, emphasis in original)

After having thus introduced the parity principle, the authors present the core of the hypothesis, the coupled system claim (see p. 2), and add:

All the components in the system play an active causal role, and they jointly govern behaviour in the same sort of way that cognition usually does. If we remove the external component the system’s behavioural competence will drop, just as it would if we removed part of its brain. [...] The external features in a coupled system play an ineliminable role – if we retain internal structure but change the external features, behaviour may change completely. The external features here are just as causally relevant as typical internal features of the brain. (Clark and Chalmers 1998, 8 f)

If, taking the first claim at face value, a part of the world worked as a process that could, in practice or in principle, also occur in the head, then the ‘external’ and the ‘internal’ versions of that process would be equivalent, hence interchangeable without loss, and arguably without a difference beyond the physical location of that process. If however, according to the second claim, we took some environmentally coupled cognitive process and removed the external component, we would *not* be left with an intact set of internal operations within the person accomplishing the cognitive task—only minus that component. Even less would we be left with a set of internal operations identical to the case of accomplishing the same task by internal means. As Clark and Chalmers implicate in the second passage, very different kinds of mental operations are required for doing a calculation in one’s head and for doing it with a pocket calculator, or for finding one’s way by visual recognition of a scene or by consulting a map or a notebook.

Clark and Chalmers’ choice of examples is not precisely helpful in keeping these points apart. The examples they extensively discuss in order to substantiate their hypothesis—which are the examples that also dominate the Extended Mind debates—concern devices that we are suggested to consider as substitutes for, or supplements to, internally based cognitive processes. Their notebooks, keyboards and pocket calculators are artefacts that can be used for operations that could be, at least prima facie, equally accomplished ‘in the head’, for example making a calculation or remembering an appointment or the directions to a certain place.

This choice of examples not only seems to suggest ${\textit{Int}}_{W}$ but also, however inadvertently, creates the impression that the extensions in question might be mere instruments of cognition—which is a logical *non sequitur*: while ${\textit{Int}}_{S}$ stands in a relation of mutual implication with the constitutiveness claim $({\textit{Ext}}_{C}),$ the instrumentalist claim $({\textit{Ext}}_{I})$ and ${\textit{Int}}_{W}$ are logically independent. The claim that entities in the environment *might* be viewed as parts of cognitive processes does allow for the possibility but does not necessarily imply that these entities will work as instruments of a cognitive process, nor does the reverse hold. At the same instance, one will be entitled to accept that ${\textit{Ext}}_{C}$ and ${\textit{Ext}}_{I}$ might hold at the same time, albeit in different, non-intersecting domains: some extensions might play a constitutive role, and thus imply ${\textit{Int}}_{S}$ for the respective class of cases, while others might count as mere instruments of cognition in another class of cases. Still, ${\textit{Int}}_{S}$ will be vindicated, since there *is* a class of cases by which it is implied. On this analysis, the ${\textit{Ext}}_{C}$ type of cases provides much better support for the Extended Mind Hypothesis, and it does so for its stronger version.

In conjunction with the aetiological argument, these distinctions will contribute to a reasonably detailed classification of relations of extendedness. Any claim for a functional parity between the internal and external mechanisms involved will have to be cashed out in some, not necessarily directly biological, reproductive advantage determinable for each part of the equation.

If, firstly, I were able to substitute an environmentally coupled for an internal process at will, where that substitution made no difference to the achievement of my goals, in terms of scope, effectivity, efficiency or reliability, the functional equivalence involved would be of the weak ${\textit{Ext}}_{I}$ kind. We still would have to explain how the elements involved have come to be thus substitutable.

If, secondly, the members of some population become equally able to mobilise some novel environment-based mechanism in order to supplement an established cognitive function, so as to achieve an increase in scope, effectiveness, efficiency or reliability, functional equivalence of a non-trivial constitutive$_{W}$ kind will be reached, while adaptive fitness will actually be increased across the entire population. This is where some of the standard examples of cognitive extensions will fit in, namely computing devices that increase the amount and complexity of available information, or the speed of retrieval.

If, thirdly, members of some population become able to individually mobilise a novel environment-based mechanism for an established function, so as to outperform other members of that population to whom the mechanism remains unavailable, the condition of functional equivalence will hold with respect to the sameness of function but not with respect to adaptive fitness within the population. Such would be the case for technologies of cognitive enhancement that are only available to some members of a population.

If, fourthly, I use an environmentally coupled process in order to compensate for an internally based one that I am not able to perform for some reason, and if that environmentally based substitute enables me to accomplish the same task with roughly the same degree of effectivity, efficiency and reliability (see Clark’s “glue and trust” criterion, p. 7), functional equivalence will be reached on all levels by means of a mechanism that is clearly distinct from the impaired one. Such will be the case in what has been termed “sensory substitution” (see Farina 2013; Kiverstein et al. 2014): situations of sensory impairment might be mended by artefacts that compensate for the function that has been lost, and that typically recruit an alternative sensory modality.

If, fifthly, an environmentally coupled process allows for the establishment of novel functions, and hence is constitutive$_{S},$ there will be no reference point for determining functional equivalence, so that the function in question will have to be identified in the first place. It is upwards along this hierarchy of types of functional relations that one will find the most rewarding proving grounds for any claim of extendedness.

## Constitutional matters

The distinction between constitutive$_{W}$ and constitutive$_{S}$ types of cases introduced in the previous section partly matches the respective foci of “first wave” parity-oriented views of extended cognition, which highlight functional equivalence between internal and environmentally coupled cognitive processes, and “second wave” complementarity views, which highlight the role of environmentally extended processes as co-constituents of some cognitive abilities.^11^ Still, the mapping of these “waves” onto the types of constitutiveness introduced above is not strictly disjunctive, and some of the preceding points might help to partly integrate these arguments: where classical functionalism focused on analogies between input/output relations in different systems on a general level, and where the “microfunctionalism” defended by Wheeler (2010) focuses on more fine-grained analogies in functions of the structures that realise a superordinate function, a history of selected effects of the traits of the systems under consideration will help to explain the establishment of certain functions in the first place. At the same instance, it is a matter of course to an aetiological view of functions to be impartial towards somatic boundaries, provided that the environmental structures in question form part of the necessary conditions in an explanation of the presence of some function. It is equally a matter of course for an aetiological view, as it is for the developmental systems theorist, to accept that the roles played by the co-constituents of some function are more likely heterogeneous in kind than mutually substitutable.

Still, one might accept either or both types of constitutive roles of environmental factors for some of the adaptive functions of some organism’s traits without necessarily accepting them as being coupled with the organism in the sense suggested by Clark and Chalmers. According to Sterelny (2010), the Extended Mind Hypothesis overemphasises prima facie functional similarities between internal and artefact-involving cognitive processes while playing down the role of the agents who not only rely on environmental supports but actively mobilise environmental resources, natural and other, so as to construct their own cognitive niches (a concept more richly developed in his 2003 book). Sterelny (2010) argues that functional couplings of the Extended Mind kind are limiting cases of the more general phenomenon of environmental scaffolding: the reliance of organisms on the presence of some environmental features or artefacts without which some function could not be accomplished. For example, human beings have become reliant on the availability of cooked food or means of cooking food, as their metabolic apparatus has become insufficient for coping with exclusively raw food. Hence, cooking is an environmental scaffolding for the human metabolism, but it will be hardly illuminating to refer to the human metabolism as being extended into the environment.

With respect to constitutive$_{W}$ cases, all hopes for an artefact to pass muster as being part of an extended cognitive processes rather than a mere scaffolding rest on the degree of integration of the artefact into the accomplishment of a cognitive function, such as memorising facts or orienting oneself in space—which are functions that still can be accomplished to some extent by the unaided mind. However, genuinely material “anchors” for certain cognitive tasks may often be both more efficient and historically prior to analogously formed conceptual ones (Hutchins 2005). Still, there will always be room for debate as to whether some concrete coupling with some artefact is tight and integrated enough to warrant the bestowal of the “extended” predicate, or to make us, in Clark’s words, “natural-born cyborgs” (2003). There is no steadfast ontological criterion to make that decision for us, although the evolutionary history of the mechanisms involved will provide some guidance.

Hence, the quest will be for evolved mechanisms for recruiting artefacts for the performance of cognitive functions. Clearly, it is not some concrete type of artefact (the notebook, the smartphone) that we have evolved to be coupled with. What can be credibly argued to have evolved is a general and highly adaptable ability to create and recruit such artefacts—from simple drawings to computing gadgets. In Millikan’s terms (see p. 5 above), it is the direct proper function of the organism-based mechanisms involved to recruit a variety of artefacts for the accomplishment of a cognitive task, and it is the direct proper function of any concrete type of artefact involved to make its contribution to the accomplishment of that task, in co-operation with the organism-based mechanisms. If, though, one takes the lessons to be learned from developmental systems theory seriously, the practice of using artefacts should be expected to feed back into the organisation of the brain, and might do so beyond childhood development (Farina 2014).

It will be more difficult to identify the nature and functions of instances of environmental coupling that play a constitutive$_{S}$ role, as the respective cognitive ability supposedly would not exist without the environmental counterpart. It will even be difficult to identify the ability in question as being properly *cognitive*. If one follows Adams and Aizawa (2001) in defining cognitive processes ab initio as a subset of the processes within the human nervous system, one has a criterion for what counts as cognitive that rules out “extra-cranial” processes by default, albeit a question-begging one. After all, there are many non-cognitive neuronal processes. It is the additional criterion of the involvement of “non-derived content” recruited by the authors that shall suffice to identify the subset of intra-cranial processes that are properly cognitive. In introducing that second criterion, the authors follow an argument in Searle’s critique of Artificial Intelligence (1980): while linguistic items or other artefacts have the meanings they have and serve the functions they do because they are endowed, by stipulation or convention, with those meanings and functions, our thoughts have their meanings and serve their functions in at least partial independence from any such external fiat. The nature of the human mind bearing those thoughts is supposedly at least partly sufficient for the presence of that content. That nature is not further explicated though.

On the aetiological view, in contrast, both artefacts of all sorts and our thoughts have the meanings and functions they have because individual artefacts and mental tokens have been reproduced by some mechanism with sufficient frequency because they mapped onto some world affair, in terms of being connected to an appropriate set of behaviours towards it, reliably enough and according to a well-defined set of regularities. On this view, cognition basically is a specific activity of relating to one’s environment, in which such mapping relations are used to produce adaptive behaviours. No principled difference exists between artifactual and internal mechanisms that accomplish this feat, namely producing the appropriate tokens on the appropriate occasions, where the conditions of appropriateness essentially depend on what in the environment the organism relies on. As we will see in more detail in the next section, the human faculty of language is the primary candidate for a properly cognitive trait that may both count as environmentally extended *and* fits the constitutive$_{S}$ condition, and hence militates against either of Adams and Aizawa’s criteria: its function and contents depend on the operation of mechanisms that partly operate outside the human brain or body.

A more detailed attempt at spelling out what kind of specific activity of relating to one’s environment cognition is has been undertaken by Godfrey-Smith: if “the function of cognition is to enable the agent to deal with environmental complexity” (1996a, p. 3, the “environmental complexity hypothesis”), the criterion for a process to count as cognitive is whether it contributes to dealing with such complexity. Cognition has a function if an environment is distally complex and changeable but, at the same instance, correlated with proximal, detectable conditions reliably enough to warrant the effort of tracking the latter, so as to modify one’s behaviour towards the former. If proximal conditions in one’s environment were too chaotic, resilience would be the preferable evolutionary strategy instead. If, in turn, distal conditions were too dull, local confinement to an optimal range of conditions would be the solution of choice. Neither perception nor object recognition nor memory would be required in these latter two types of cases, but they will be essential to the first one. On this analysis, cognition arises with the necessity of either tracking favourable conditions or actively making conditions favourable (see p. 10).

Entities and processes in the environment may contribute to these abilities in constitutive$_{S}$ fashion in either of two ways, both of which include evolved mechanisms of coupling: on the one hand, they might be part of the necessary conditions for the realisation of the direct proper functions of a mechanism that is otherwise based within the organism. The entities and processes in the environment need not be reproducible items with their own direct proper functions, but their presence and use will be essential to the realisation of the functions of the organism-based production mechanisms. On the other hand, there might be mechanisms for the production of tokens of some type that are based in the environment while being co-ordinated with organism-based mechanisms, so as to jointly help him to produce behaviours appropriate to a given world affair in a given situation. It is in the latter, more complex type of case where reproducible artefacts will come to play a central role.

## The art of coupling, basic and advanced

Clark and Chalmers only briefly and tentatively refer to relations that would count as constitutive$_{S}$ on the present account (1998, p. 11). However, these references are part of a brief evolutionary excursus and include two suggestions that match the distinction introduced at the end of the preceding section: the evolution of vision as exploiting the structure of local environments, and the evolution of language as a key structuring feature of human interaction.^12^ While Clark and Chalmers proceed to using language as their paradigm extension of the mind, it might be worthwhile to pay attention to more basic modes of environmental coupling. The function of vision and language is certainly not one of instruments that could be used or discarded by human beings at will. In the context of human evolution, both are indispensable components of, and hence constitutive$_{S}$ to, human cognition. If an individual is deprived of either faculty, his or her cognitive functions will be impaired to some extent, and some functions could not be accomplished at all.

Still, there is a difference between vision and language, with their respective modes of environmental coupling, in terms of the depth of their evolutionary roots and the levels of cognition they feed into. Vision is not only the older and more common phenomenon in the animal kingdom. It can also be plausibly argued to be more directly tied to objects and structures in the environment, serving as a prerequisite for spatial orientation and object recognition for most animals, including humans. Language, in contrast, serves the coupling between human agents in the first place and presupposes a number of pre-linguistic cognitive capabilities, including mechanisms of orientation and object recognition. Moreover, whereas the environmental coupling in visual perception, in terms of necessary constituents of the visual process, occurs between mechanisms of perception and invariant properties of objects in the environment, language will rightfully count as a production mechanism in its own right.

On this view, the cognitively most foundational set of external entities to be coupled with is to be found in natural objects or processes in the environment. According to the ecological view of visual perception, as developed by Gibson (1979), features of an organism’s natural environment will guide his activities and be available to him reliably, directly, implicitly and automatically, without the need of reflection upon their operation, and without the need of having been *consciously* endorsed in the past. Reliably successful endorsement in the past will be necessary and partly sufficient. Thus, all of Clark and Chalmers’s criteria cited on p. 8 above will be met.

More precisely, the Gibsonian will hold that a perceiving organism is engaged in the activity of picking up information from his environment, where invariants in the structure of that environment are the bearers of that information, and where the organism’s own position, movements and orientation in relation to some object—and, as Lauwereyns (2012) emphasises in his ecologically minded but neuroscientifically based “intensive” account of vision, his purposes towards that object—will be as essential to the structuring of perception as those invariants. The object will then quite directly offer or “afford” possible behaviours to the organism, depending on how he is disposed and positioned towards it. Visual perception is not to be conceived of as a string of retinal images passively received in order to be processed by the perceiver’s nervous system. It crucially relies on the perceiver’s interaction with his environment. On the ecological view, inference or inner representations are largely irrelevant to perception, whereas none of these higher-order cognitive accomplishments could emerge without the prior establishment of mechanisms of perceptual coupling. Alternatively, between such elementary direct guidance by perception and fully fledged internal representations, one might find representations that detect patterns in one’s environment and direct appropriate behaviours towards them at the same instance. These elementary representations have been introduced as “pushmi-pullyu representations” by Millikan (1995), and they figure as “action-oriented representations” in Clark (1997; see also Clark and Toribio 1994).

This image of directness has to be extended when moving from perception to higher-order kinds of cognition. On the one hand, some of the guidance that has hitherto been directly provided by perceptual couplings with the environment is now delegated to internal resources, some but not all of which will assume the shape of explicit representations. Thus, the next remarkable development, again in phylogenetic terms, does not as much lie in an extension of cognition into the environment as it lies in the internalisation of some of the guidance that would otherwise be directly provided by the environment. Externalisation reappears on the level of cognitive artefacts, that is, devices that are used for cognitive functions, thus re-instantiating environmental guidance on a higher level.^13^

On the other hand, the purposeful creation and appropriation of cognitive resources fosters their (partial) detachment from a present, imminent or otherwise pre-determined practical use. In turn, however, the use of these artificial resources, once it has been properly learned, and if it functions smoothly, *normally* is quite as direct, implicit and automatic as in the more basic cases. One may pause to reflect on the information taken up from the environment, and one may then either resort to other sources of information or store away that information for later use. However, doing so remains a special case mostly reserved for instances of interference or error, and for (re-) assessing the goals of one’s actions. In a familiar and well-behaved environment, the natural and artificial features of that environment will equally provide direct guidance.

Language has the intriguing property of essentially involving both artefacts and internal mechanisms in order to perform its functions. It is at once rooted in evolution and in the history of artefact use. More specifically, it has been argued, most prominently by Deacon (1997), that language co-evolved with the human brain and its capability of symbolic reference. Deacon takes language to be a structure whose properties and reproduction within language communities are in a straightforwardly evolutionary way interdependent with the properties particular to the human brain. The complexity and the adaptive functions of the human mind have as one of the necessary conditions of their emergence and present functioning the development and use of linguistic structures. The intra- and extra-somatic mechanisms for the production of linguistic items are tightly integrated with each other. Neither mechanism would be present nor could not function in absence of the other. Individually, they contribute to shaping their counterpart and its functions, historically and at present. Jointly, they enable speakers to create concrete artifactual structures on which their further interaction relies. This claim is to be distinguished from the considerably broader view that human linguistic capacities are a product of natural selection on the one hand, where the possible role of linguistic artefacts in shaping these capacities is not further considered, and Chomskyan linguistics on the other, where linguistic structures proper are supposed to be at once innate and *not* a product of natural selection.^14^

If the human faculty of language and linguistic structures in conjunction are thus correctly described as achieving a complex cognitive coupling between agents and their environments that would not be possible without them, they may actually and at the same instance be the cognitive resource with the highest potential for *de*coupling, as they allow for public *and* individual use in a multitude of, partly unanticipated, contexts, and as they allow for degrees of abstraction and detachment from actual matters of fact that would otherwise be hard to come by. Hence, contrary to one concluding observation in Clark and Chalmers’ essay, the human mind, without language, would *not* be “much more akin to discrete Cartesian ‘inner’ minds, in which high level cognition relies largely on internal resources” (1998, p. 18). If we may call our evolutionary kin as witness, our minds would be coupled with their environments in considerably tighter and more direct fashion, in that there would be little exception to being directly guided in our actions by what we encounter there.

## Conclusion

If constitutive modes of cognitive coupling carry the day as the paradigms of extensions of the mind, as I have sought do demonstrate, we might better conceive of the human and other minds as extended not by virtue of being equipped with a number of artificial add-ons that work as external instruments of internally based cognition. Instead, we should conceive of cognition as being extended over traits of the organism and properties of the environment, incorporating and integrating entities, processes and mechanisms on either side of the somatic boundaries. Both sides have evolved, grown and, in part, purposefully created to be so integrated. In conjunction, they will either constitute$_{W}$ alternative mechanisms for the accomplishment of some cognitive functions or constitute$_{S}$ some functions, as necessary conditions for their presence. In either case but to different degrees, the establishment and performance of these functions depends both on evolved mechanisms and on the environment-shaping activities of the cognising organisms themselves.

To end on a historical note: whether developmental systems theory or environmental constructivism or niche constructivism or teleofunctionalism or theories of extended cognition are concerned, they appear to share a leitmotif introduced into modern Anglo-Saxon philosophy by Dewey (especially in 1929).^15^ In critical reaction to Herbert Spencer’s self-styled Darwinian conception of the organism–environment relation and in accordance with a different lesson drawn from Darwin, Dewey considers the distinction between organism and environment artificial to begin with. Presuming that distinction to be ontologically grounded is likely to create many of the problems that most accounts of the adaptive efforts of an organism keep struggling with. To live plainly *is* a particular way of being related to one’s environment. It also is an interactive, environment-shaping relation, and it is continuous with being a cognitive system, in terms of the evolved function of cognition to help an organism cope with a complex and continuously changing environment. On this view, it will be difficult to find cognitive processes that do not incorporate features of the environment. At root, all cognition is extended, and only some of the historically most recent cognitive traits allow for a notable degree of decoupling. The mind is not extended *into* but *over* the environment.

## Footnotes

1 For these, historically prior, kinds of externalist view, see Putnam (1975a) and Burge (1979) in philosophical semantics and McGinn (1977) in the philosophy of mind.

2 For concise attempts at systematically mapping that debate, see Menary (2010b) and Wilson and Clark (2009). Hurley (2010) embeds her account in a broader taxonomy of externalist positions in the philosophy of mind. A useful commented bibliography on extended cognition is provided by Kiverstein et al. (2013).

3 This concern is shared by the main critics of extended cognition (Adams and Aizawa 2001; Rupert 2004; Sprevak 2009).

4 See, for example, Wheeler and Clark (2008), where evolutionary and developmental considerations play a major role in an argument for dynamic interactions between genetic, organismic and cultural factors; or Wilson and Clark (2009), where an explicit and positive reference is made to evolutionary accounts of biological functions; or Rowlands (2004, pp. 224–227) who refers to Millikan-style proper functions and their aetiological credentials.

5 The *loci classici* for this distinction are Owen (1848, p. 7) and Darwin (1859, p. 434).

6 Other paradigmatic statements of this approach include Papineau (1987), Neander (1991a, b), Godfrey-Smith (1994) and Price (2001); see also the anthology by Macdonald and Papineau (2006).

7 For the full definition of proper functions, see Millikan (1984, p. 28 and Chaps. 1–2 in general).

8 Remarkably, this link between teleosemantics and extended cognition was first highlighted by one of the main critics of the latter: Rupert (2004, p. 401, n22).

9 At the end of (2008), Clark asks for a “mental flip” explicitly styled after the one suggested by Dawkins (1999), whereas much of the preceding text is an attempt at providing concrete evidence for the explanatory value of extended cognition.

10 Among the few non-primate tool-using species, individuals of the New Caledonian crow (*Corvus moneduloides*) were observed to alternate between tool-guided and tool-less techniques of extracting food in the wild (Bluff et al. 2010). A recent example of allegedly innovative tool-use and its social transmission in captive Goffin cockatoos (*Cacatua goffini*) is to be found in Auersperg et al. (2014). For an authoritative account of the varieties and complexities of animal tool use, see Shumaker et al. (2011).

11 The distinction between waves of arguments for extended cognition has been made by Sutton (2010). Defences of extended functionalism include Clark (2008, 2010b) and Wheeler (2010), while second wave views have been introduced by Menary (2010a, integrationism), Sutton (2010) and Kiverstein and Farina (2011, complementarity), and Rowlands (2010, amalgamationism). It was Clark (1997) himself who anticipated complementarity views.

12 For extended-mind-based accounts of language, see Clark (1998, 2006) and Wheeler (2004).

13 The term “cognitive artefacts” is not used by Clark and Chalmers (1998), but can be found in Clark (1998). Sterelny (2004) uses the roughly synonymous term of “epistemic artefacts”, which in turn is taken up by Clark (2010b).

14 See Christiansen and Kirby (2003) for a collection of positions on the evolutionary roots of language and the possibility of brain/language co-evolution. For a classic, anti-Chomskyan account of the evolution of the human faculty of language, see Pinker and Bloom (1990), and for the position under attack here Chomsky (2006).

15 For historical and systematic considerations of the Spencer–Dewey axis, see Pearce (2014) and Godfrey-Smith (1996a, b). Notably, Dawkins’ extended phenotype theory may be considered playing the Spencerian part in this context.
